# Understanding Environmental Pollutions of Informal E-Waste Clustering in Global South via Multi-Scalar Regulatory Frameworks: A Case Study of Guiyu Town, China

**DOI:** 10.3390/ijerph17082802

**Published:** 2020-04-18

**Authors:** Kun Wang, Junxi Qian, Lixiong Liu

**Affiliations:** 1 Department of Geography and Resource Management, The Chinese University of Hong Kong, Shatin, New Territories, Hong Kong, China; Kungle250@gmail.com; 2 Department of Geography, The University of Hong Kong, Hong Kong, China; jxqian@hku.hk; 3 School of Architecture and Urban Planning, Guangdong University of Technology, Guangzhou 510006, China

**Keywords:** informal e-waste recycling, environmental pollutions, geographical clustering, multi-scalar regulatory frameworks, China

## Abstract

The recycling of e-waste by the informal sector has brought countries in the Global South raw materials (e.g. metals and plastics), second-hand electronic equipment and components, and economic opportunities in conjunction with appalling environmental pollutions and health problems. Despite the longstanding international and national legislation regulating transnational trade and domestic recycling, informal e-waste economies are still clustering in many Global South countries. This study offers historically and geographically specific explanations of this conundrum, by interrogating the multi-scalar regulatory frameworks in which the informal e-waste economies and their pollutions are embedded, by drawing on China, particularly the former global e-waste hub-Guiyu town, as the case study. We argue that the contested and problematic application of current international and national legislation in regulating e-waste is in part pertaining to the slippery definition of what counts as “e-waste” and its paradoxical nature as both resources and pollutants. At the global scale, trajectories of global e-waste flows are shaped by the multitude of loopholes, contradictions and ambiguous articles left by the Basel Convention and by different countries’ disparate attitudes towards the e-waste trade. At the national scale, the ambiguities and contradictions in the Basel Convention have been passed on to and shaped China’s national e-waste regulatory frameworks. China’s equivocal legislation, paradoxical attitude, and formal enterprises’ weak competence contribute to the rise of informal e-waste recycling in Guiyu. Yet, China’s e-waste regime has been greatly restructured within the past decade, with formal recycling enterprises playing an increasingly significant role.

## 1. Introduction

Over the past half century, swift technological advancement in the electrical and electronic industry has revolutionized the world. The amount of electronics has been increasing at an astonishing speed in both developed and some newly industrialized countries (e.g., China and India) [1]. The public, often perceiving the electronics industry as a “green” and high-tech sector, rarely associates it with environmental pollutions and toxics. Nonetheless, proper and safe disposal of e-waste requires special devices and techniques, marking off e-waste from other types of municipal waste. Despite the considerable quantities of valuable materials contained in e-waste, current techniques and management models have limited capability to recycle them, given the wide range of e-waste categories, the complex physical design and chemical composition, and the hazardous components; thus, only a very small proportion of e-waste has been properly handled without producing environmental pollutions and health problems [2].

A substantial proportion of e-waste produced in developed countries has been transferred to some developing countries, dismantled by low-skilled labors in the informal sector. The informal e-waste processing in these countries has a territorial tendency, resulting in alarming environmental pollution and health risks at the processing locus. Many geographical clusters specialized in the informal recycling of e-waste in developing countries have been reported by mainstream media and environmental NGOs. The Guiyu town in China, before the government’s recent crackdown, was one of the largest informal e-waste processing clusters in the world, processing millions of tons of e-waste annually, most of which were from developed countries [3]. As such, Guiyu was nicknamed by the media and environmental NGOs in many occasions as the “electronic graveyard of the world”. Varied forms of primitive techniques have been employed by recyclers at these informal clusters to recover useful materials from e-waste. Among these rudimentary methods, open burning is most environmentally detrimental, followed by mechanical treatment and leaching [4]. Very high levels of heavy metals (e.g., lead, cadmium, mercury) and organic pollutants (e.g., polychlorinated biphenyls—PCBs; polybrominated dibenzo-p-dioxins/dibenzofurans—PCBB/Fs) have been detected in air, water and sediments in these informal e-waste clusters (ibid.) Variegated health problems pertaining to informal e-waste recycling (e.g., blood lead poisoning, cancer, miscarriage) has been reported by environmental and health specialists (ibid.)

The cross-border transfers of e-waste from developed countries to developing countries, in most cases, have been rendered illicit by both international treaties and China’s national legal provisions that have existed since the 1990s. China banned the import of e-waste in the early 2000s. However, according to environmental NGO’s investigations, large quantities of e-waste were still entering, with an estimate of between 60 and 90 percent of the globally produced e-waste being illicitly traded or dumped [5]. As such, the primary research question of this paper is concerned with the policy and regulatory dimensions of the state, i.e., why and how can e-waste still ‘flow’ to informal e-waste clusters in countries like China, despite the longstanding international and national legislation? Besides, of late, the import of e-waste has declined substantially in China, so our second question is, what are the new changes and trends of China’s e-waste regulatory regime? To date, most of the research in the field of e-waste has been dominated by the “hard science” circles, for example, environmental science, public health, waste management, engineering, chemistry, etc. and have provided valuable scientific explorations for the public to understand the specific environmental toxicities and health problems associated with e-waste, Whereas there are few social scientific explorations of multi-scalar e-waste regulatory frameworks. As such, this study contributes to the literature by offering a systematic analysis of the multi-scalar e-waste regulatory frameworks, to understand why and how illegal flows of waste can persist (flows from developed countries to informal e-waste processing clusters like Guiyu in China), despite the fact that both international and national legislations prohibit it. This paper, therefore, is basically a policy research study, drawing heavily on policy documents and rationales, both at the national and transnational scales, but also paying special attention to policy practices at the local scale of Guiyu. Above all, it connects the complexities, paradoxes, and ambiguities of the multi-scalar regulatory frameworks to the ambivalent nature of e-waste, as both resources and hazardous waste/pollutants.

This paper is structured as follows. It first introduces the methodology in Section 2. Then, it provides the systematic documentation of the multi-scalar e-waste economies and its geographical clustering, by tracing the global production, transnational flows, domestic generation of e-waste and its informal recycling in Guiyu (Section 3). After that, it explores how these multi-scalar e-waste space-economies described in Section 3 are respectively embedded in the global/international regulatory frameworks (in Section 4) and the national regulatory frameworks (Section 5), before making the conclusion remarks (Section 6).

## 2. Methodology

The research questions, which entail multi-scalar analyses, cannot be answered if one simply focuses on either Guiyu or China as the unit of analysis. As a result, this paper answers the research questions by unravelling the multi-scalar e-waste regulatory frameworks, in which the agglomeration of informal e-waste processing in Guiyu is embedded. Methodologically, it draws on policy analysis, case study, ethnographical fieldworks, and grey literature, consisting mainly of media and NGO reports.

First, in respect of policy analysis, it conducts a meticulous examination of e-waste regulations and laws at different scales, to explore how they shape the transnational e-waste flows and informal e-waste recycling in China. At the global scale, the Basel Convention on the Control of Transboundary Movements of Hazardous Wastes and Their Disposal (the Basel Convention, hereafter) make up the global legal infrastructure addressing the transnational trade of e-waste. It is the centerpiece of an international legal regime that has shaped or influenced many countries’ national legislation on e-waste. Therefore, the provisions, annexes, and subdocuments of the Basel Convention are carefully examined. The analytical results reveal a multitude of loopholes, contradictions and ambiguous provisions in the Convention, that make it difficult to apply the convention to constrain e-waste from developed countries to developing countries. Some other international e-waste regulations (e.g., the e-waste regulations of the European Community and the US) that have disparate impacts on the export of e-waste to China are analyzed as well. At last, China’s national regulatory frameworks of e-waste and Guiyu’s local policy guidelines are scrutinized.

Second, this research uses an informal e-waste processing cluster, Guiyu town in China, as the study case to explore the multi-scalar regulatory frameworks of e-waste. Before the recent tightening of e-waste import, China had been the largest receiving country of e-waste from developed countries [6]. Many informal e-waste processing clusters have been founded in China. Guiyu in China and the Agbogbloshie community of Accra in Ghana are the largest two informal e-waste recycling hubs in the world, receiving millions of tons of e-waste from developed countries [7,8]. China has a large informal sector specializing in e-waste import, trade, collection, and recycling [9]. More recently, China itself has taken the place of the U.S. as the largest e-waste-generating country in the world [1]. A large amount of the increasing domestic e-waste has been transferred to Guiyu as well. As such, Guiyu in China is an exemplar case for studying the multi-scalar regulatory frameworks of e-waste.

Third, given the murky legal status of informal e-waste trade and processing in China, ethnographical fieldworks and the grey literature can provide valuable information alongside the policy analysis. The ethnographic fieldwork was carried out between October 2016 and September 2018 in Guiyu, to achieve grounded knowledge of the practice of informal recycling shaped by the multi-scalar regulatory frameworks. More than 80 e-waste recyclers, 32 government and village officials, and many local people and rural migrants in Guiyu were interviewed. Hundreds of e-waste reports from both the international and Chinese media and international environmental NGOs (such as Greenpeace) are carefully collected and analyzed. The empirical analyses are based on the above-mentioned sources, unless specified.

## 3. Multi-Scalar Informal Economies and Geographical Clustering

### 3.1. Global Production, Transnational Flows, and Territorial Agglomeration of E-Waste Recycling in the Global South

Given the vast quantity of annual generation, e-waste has become a global concern over the past two decades. The estimated number of PCs existing across the world is more than two billion [10]. More than 3.8 billion units of electrical and electronic equipment, including 265 million PCs, were sold in EU countries in 2009 [11]. An estimate of more than 40 million tons of e-waste is produced around the globe annually (see Table 1), with a yearly growth rate of approximately 5 to 10 percent [12]. On the whole, electronics are primarily consumed and discarded in developed countries and some newly industrialized countries. In the year 2016, the average generator of e-waste per inhabitant was 20 kg/inh in North America, 17.3 kg/inh in Oceania, 16.6 kg/inh in Europe, 4.2 kg/inh in Asia, and 1.9 kg/inh in Africa [1]. Approximately 8.3 to 9.1 million tons and more than 6 million tons of e-waste were generated in 2016 in the EU countries and the U.S., respectively [1,2]. More than 500 million computers became obsolete between 1997 and 2007 in the U.S. [13] Nevertheless, only less than 10 percent of the globally produced e-waste is recorded to be formally collected and properly recycled [1].

Despite the enormous generation, e-waste has been banned from landfills in many developed countries since the 1980s [11]. Pertaining to the strict environmental regulations and high labor cost in developed countries and the increasingly declined transnational transport cost [14], industrialized countries tempted by economic interests usually tend to export them to South countries, mainly China, India, Ghana, Pakistan, Nigeria, Vietnam, and the Philippines, rather than handle them in home countries [12,15]. The cost of processing e-waste in America is ten times more expensive than shipping them to Asia [3]. According to NGOs’ investigation, roughly 50 to 80 percent of the e-waste collected by the U.S. recycling sector, the former largest e-waste generator, was exported to Asian countries, 90 percent of which was shipped to China [16] before China’s recent tightening of e-waste import. Moreover, e-waste processing activities are booming in some African countries in recent years, due to the latest tightening of regulations in Asian countries [1]. Approximately 85 percent of the e-waste shipped to Ghana and 75 percent to Nigeria are from EU countries [17].

The wholesale e-waste has been transported to developing countries through some major global transport hubs for cargo, through the collaboration between e-waste recyclers in developed countries and those e-waste brokers from informal e-waste processing sites like Guiyu in China. Hong Kong, Singapore, Taiwan, and Dubai are the common transfer stations for the global e-waste flows. For instance, in the Northern suburb of Hong Kong, e-waste brokers hired low-skilled labors conducting a primary dismantling to destruct e-waste into different components. These components were then mixed with other metal scraps (such as copper) that could be used as raw materials to be shipped into China (Metal scraps that could be used as raw materials were allowed to be imported into China until 2017, while e-waste was not). In addition, a substantial amount of e-waste had been transported into China through the border between Guangxi Province and Vietnam. After e-waste arrives in countries like China, its treatment is handled mainly by the informal sector, which usually operates outside national regulations. In most developing countries, there is a lack of insufficient formal facilities processing e-waste. Hitherto, 50 to 80 percent of the e-waste in these countries (including both domestically generated and imported) is recycled by informal recyclers [8]. For instance, India has only three formal recyclers [19], with 99 percent of its e-waste ending up in the informal sector [20]. More than 1 million poor people in India are engaging in informal e-waste recycling (Pinto, 2008). Approximately 70 percent of the e-waste arriving in New Delhi comes from developed countries [11].

A multitude of informal e-waste processing clusters in some developing countries other than China have been reported by research reports, academic papers, and the media. E-waste scraping yards have been found in Meerut, Ferozabad, Delhi, Chennai, Bangalore, and Mumbai in India [6]. In Dhaka, roughly 60,000 people are engaged in e-waste recycling activities in Elephant Road, Gulisthan, Motijheel, and Kotwali [21]. In Vietnam, informal e-waste disposal is very intensive in Trang Minh, Dong Mai, and Bui Dau [22]. In Africa, Nigeria is the largest e-waste importing country, and nearly all of the e-waste flows into the informal sector [6]. Monthly estimates of e-waste shipments to Ghana range from 300 to 600 40-foot-long containers (each with 2390 cubic feet of storage space), arriving at the port of Tema [7]. E-waste recycling sites are also found in Abidjan of the Ivory Coast. In these clusters, recyclers generally depend on rudimentary equipment and techniques. The global transfer of e-waste and the appalling environmental pollution and human health risks in these locales have been widely exposed to the public [23].

### 3.2. China’s E-Waste Production and Informal Space-Economies of E-Waste Recycling

The sources of e-waste recycled in China comprise two large parts, i.e., domestic generation and importation. First, China, as the former largest e-waste recipient, was frequently described as the “heaven of e-waste”. According to e-waste recyclers in Guiyu, most of the imported e-waste is from developed countries, including both western countries and the Four Asian Tigers. E-waste started entering China in the late 1980s. Although China signed the Basel Convention and enacted an official ban on e-waste import with the arrival of the new millennium, e-waste still found its way into the country through illegal channels, with millions of tons of e-waste smuggled into China each year (see later explanation in Section 4).

Besides, China itself has now become a main global generator of e-waste. The amount of annual domestic e-waste generation in China alone reaches approximately 7 million tons, thus overtaking the U.S. as the largest e-waste producing country in 2016 [1]. Approximately 406.8 million units of large home appliances (known as sijiyinao in Mandarin, including air conditioner, refrigerator, washing machine, TV set, and computer) (see Table 2). The total volume is also experiencing a striking growth at a rate of approximately 13 to 15 percent yearly [24]. Similarly, only around 10 percent of this domestically produced waste is handled by formal enterprises, whereas more than 80 percent ends up in the informal sector [25]. In addition, a substantial amount of e-waste has been kept at home by citizens.

The informal sector has been at the forefront of e-waste trade, collection, repair, and processing in China, with most of the e-waste, whether domestically generated or internationally imported, ending up in specialized e-waste processing sites, where agglomerated informal e-waste economies have developed. Domestically generated e-waste is usually collected by informal itinerant hawkers, who cruise different residential neighborhoods or industrial parks with a loudspeaker on their bicycles or tricycles. The domestic informal itinerant hawkers in China are usually disadvantaged social groups in cities or rural migrants who come to cities for employment. These informal itinerant hawkers are usually rural migrants who come to cities for employment. Then, the collected e-waste is selected and classified, with useful devices or components disassembled for the repair of other devices or sales in the secondary market. The remaining body is transported to e-waste processing sites. Imported e-waste, after arriving at a coastal port, is usually swiftly transferred to inland e-waste transfer hubs (e.g., Nanhai District in Foshan City, Guangzhou, Shenzhen), before being dispatched to final processing loci. Many informal e-waste processing clusters have been reported in coastal provinces or cities, including the provinces of Guangdong, Zhejiang, Hebei, and the cities of Beijing and Tianjin, although a large number of scattered dismantling sites remain unknown to the public. The alarming environmental pollutions caused by the territorial agglomeration of informal e-waste disposal in these sites create the appearance of China as the global “e-waste capital” or “e-waste heaven” in the international media or NGO reports [26].

In Guangdong Province, aside from the globally notorious Guiyu, the towns of Shijiao and Longtang in Qingyuan City, and Dali in Foshan City are hot spots of e-waste recycling as well. In Zhejiang province, many informal e-waste recycling workshops are found in the towns of Luqiao and Wenling in Taizhou City, Liushi town in Wenzhou City, and Cixi town in Ningbo City [13,14]. E-waste recycling in Luqiao emerged in the late 1970s and was taken over by Wenling after regulations in Luqiao became stricter [27], whereas both of them are overtaken by Guiyu. In Tianjin City, Ziya, a town adjacent to the Tianjin Ziya Circular Economy Industry Park (one of the largest formal e-waste recycling industrial parks in China), has agglomerated informal e-waste economies as well. Even in Beijing, the Houbajia Village and Dongxiaokou Town, located right next to Zhongguancun, which is one of the largest high-tech hubs in China and is sometimes referred to as “China’s Silicon Valley,” serve as the transaction and processing sites of China’s domestic e-waste as well. The cities of Huanghua and Wenan in Hebei Province and the Jinan District in Tianjin are also reported to have some e-waste processing sites. Thus far, a few of these sites have already been suppressed and cleaned up by the state, with many small-scale clusters remaining unknown to the public.

### 3.3. Guiyu as the Global Hub of E-Waste Recycling Economies and Pollution

**Figure 1:**
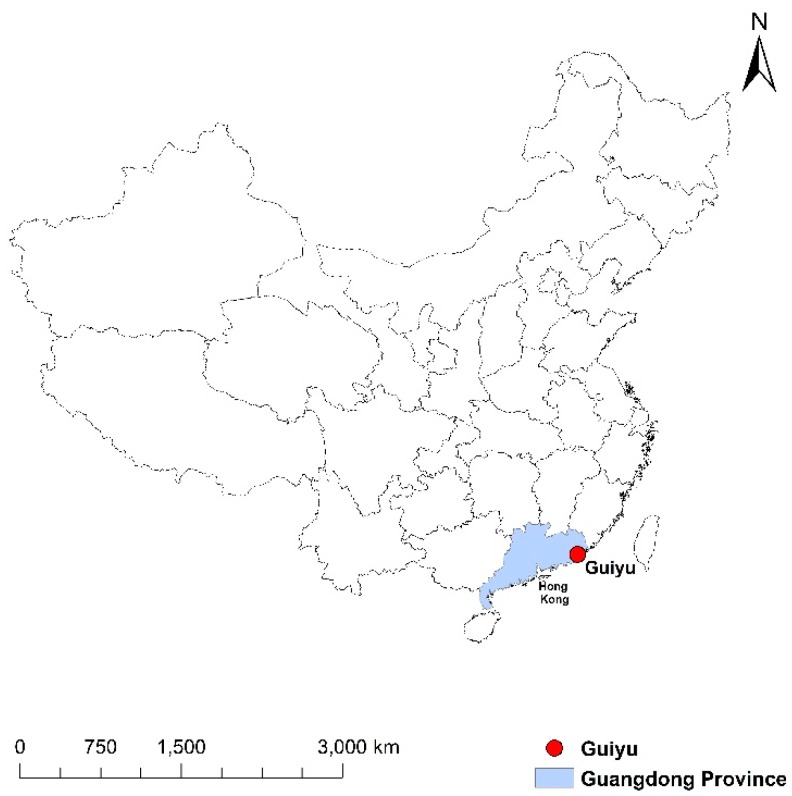
Location of Guiyu in China. (Source: Author).

Guiyu, once one of the largest global informal clusters specialized in e-waste processing before its recent formalization, is a southeast coastal town with more than 200,000 people (including both indigenous people and rural migrants) and 52.17 km^2^ land in Shantou city, Guangdong province of China (see Figure 1). With more than three decades of specialized industrial development since the late 1980s, e-waste recycling has been the pillar industry for the local economy and local people’s dependence for livelihood. According to Guiyu government’s statistics, more than 100,000 people and more than 80 percent of local families were involved in e-waste recycling in Guiyu, processing about 150 to 300 million of e-waste annually, at the peak of the industry. As a result of the appalling environmental pollutions caused by e-waste recycling exposed by international media and NGOs, China’s government implemented a large-scale formalization scheme of Guiyu’ e-waste recycling industry at the end of 2015, with many types of e-waste economies producing pollutions shut down.

**Figure 2:**
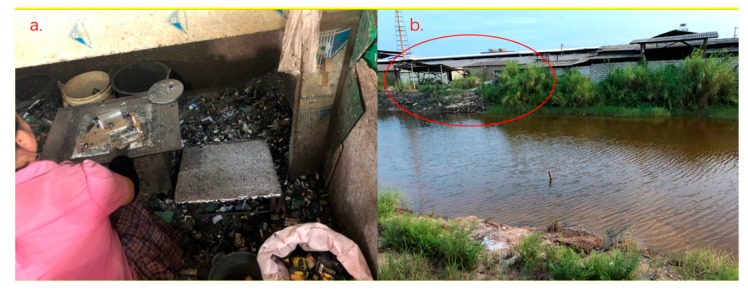
Workshops of circuit board baking and precious metal extraction in Guiyu. Source: author’s copyright.

Despite relying on low-level technologies and manual labor, a deep, subtle, and comprehensive division of labor was observed in Guiyu. Family workshops were respectively specialized in variegated types of e-waste collection, sales, processing, second-hand electronic components trade, raw materials extraction and sales. Among the variegated types of e-waste economies, circuit boards baking (CBB), precious metal extraction, and plastics disposal were the three main sources of pollution. CBB was the most important type of e-waste economy that contributes to the global infamy of Guiyu. Local people used coal stoves and liquid tin to melt the soldering points of circuit boards and taken the varied categories of useful electronic components from the board (see Figure 2a). After useful elements were taken down, the left circuit boards and those dysfunctional components would be used for precious metal extraction, which was conducted in two forms. The first was to put them into incinerators to recover valuable metals through pyrometallurgy. The second was hydrometallurgy, which was known as “gold washing” in the vernacular (Figure 2b was a gold washing workshop near riverways). Highly corrosive strong acids were used in the separation between the precious metals and the plastics contained in circuit boards. Last, the techniques mostly used for plastics disposal in Guiyu were burning and salt brine delamination. Every variety of plastic has a unique smell when it is burnt. Therefore, the recyclers ignited plastics and classified them into different categories by identifying their smell in burning. Because plastics in the varied category have different densities, local recyclers also prepared salt brine at various densities to select plastics. The chemicals used in both “gold washing” and plastics classification were directly discharged into the local waterways as well.

Guiyu has drawn widely scholarly attention of environmental and health specialists, whose research has provided scientific evidence of the environmental and health problems associated with informal e-waste recycling. Excessive concentrations of heavy metals and toxic chemicals have been detected in the soil, water, and air at Guiyu [4]. The Lianjiang River and Nanyang River in Guiyu, as a result of strong acid leaching (gold washing, as mentioned above) were respectively enriched with dissolved As, Cr, Li, Mo, Sb and Se, Ag, Be, Cd, Co, Cu, Ni, Pb and Zn [28]. Some scholars have studied the exposure levels of PCBs for local residents and e-waste workers in Guiyu, based on the PCB levels in fish (collected from local rivers), atmosphere and human milk samples [29]. Their work suggests that the homologue composition of PCBs in seven species of fish were consistent and similar to commercial PCBs Aroclor 1248; PCB levels in the air surrounding the open burning site were significantly higher than those in the residential area; inhalation exposure contributed 27% and 93% to the total body loadings (the sum of dietary and inhalation exposure) of the local residents and e-waste workers engaged in open burning respectively; the total PCB concentrations in human milk ranged from N.D. to 57.6 ng/g lipid, with an average of 9.50 ng/g lipid (ibid., 76). It is also reported that children living in Guiyu had significantly higher blood lead levels (7.06 mg/dL) than the quantity (5.89 mg/dL) of children from the controlled area Haojiang (P<0.05) [30]. The proportion of Guiyu children that have blood lead levels exceeding 10 mg/dL is 24.80%, while the figure for Haojiang children is only 12.84% (ibid.) Guiyu has witnessed significantly higher rates of adverse birth outcomes compared to the control site Xiamen, including stillbirth (4.72% vs. 1.03%), low birth weight (6.12% vs. 4.12%), term low birth weight (3.40% vs. 1.57%), and lower Apgar scores (9.6 vs. 9.9) and mean birth weight (3168 g vs. 3258 g) t, all *p* < 0.01 [3].

## 4. Global Regulatory Frameworks on Transnational Flows of E-Waste

The large quantity of international e-waste trade has given rise to the global concern on e-waste, triggering heated discussions on environmental justice and equity. As mentioned, the cross-border transfer of e-waste is usually in violation of international laws.

### 4.1. The Analysis of the Basel Convention

To date, no single international rule has been specially formulated for the regulation of global e-waste trade. The key international treaty on transnational e-waste trading is the Basel Convention, which came into force in 1992. To date, 186 states have signed the Basel Convention.

On paper, the aims of the Basel Convention are threefold: the minimization of waste and the environmentally sound management of waste; the control of the transboundary movement of hazardous wastes; and the prevention and combat of illegal traffic. The second aim is at the core of the Convention, whereas the last is affiliated to it. Yet, despite the three decades of efforts made by implicated parties, numerous issues remain and are undergoing intense debates. Based on Article 9 in the Basel Convention, any transboundary movement of hazardous wastes or other wastes without prior informed consent from importing countries will be considered illegal traffic. Nevertheless, the Basel Convention, to a large extent, remains on paper, just as the statement in the introduction of the Convention, “one of the most important contributions of the Basel Convention over the past 20 years is the elaboration of a significant number of policy instruments with non-binding character” [31]. The Convention does not impose a complete ban on the international transfer of hazardous waste. According to Article 4 (Provision 9), the transfer may be allowed in certain conditions: (a) The State of export does not have the technical capacity and the necessary facilities, capacity or suitable disposal sites in order to dispose of the wastes in question in an environmentally sound and efficient manner; or (b) The wastes in question are required as a raw material for recycling or recovery industries in the State of import; or (c) The transboundary movement in question is in accordance with other criteria to be decided by the Parties, provided those criteria do not differ from the objectives of this Convention.

The slippery definition of what counts as “e-waste” has resulted in the difficult and highly disputed use of the Basel Convention to regulate international e-waste trade. The definition of the key terms in this article, e.g., “technical capacity,” “necessary facilities,” “environmentally sound and efficient manners,” and “wastes required as raw material” can be rather controversial in practice. Different countries may understand differently what can be counted as “necessary facilities” and what varieties of materials should be seen as “e-waste.” The prescription that waste used for recycling raw materials can be exempted from the Basel Convention and makes the ban on the international trade of e-waste highly contentious. Moreover, the focus of the Basel Convention is on the regulation of the body of countries, whereas illegal waste transfer is usually conducted by private firms or individuals. At the 1995 Conference of Parties (COP.3), the Basel Ban Amendment, which prescribed that all countries listed in Annex VII (members of OECD or the European Community and Liechtenstein) should prohibit all transboundary movements of hazardous waste (including for recycling) to all non-Annex VII countries that had signed the Convention, was adopted by the Convention under the urging of Greenpeace and some less developed countries. Nevertheless, a thorough ban on the international trade of e-waste can result in the loss of revenue for some industrial advocacy groups [32]. Accordingly, due to disagreements and the lack of enough member states’ ratification, the amendment still has not come into force. Although the Protocol on Liability and Compensation for Damage Resulting from Transboundary Movements of Hazardous Wastes and their Disposal (adopted at COP. 15) is a legal document attempting to make the Convention applicable, it is accused of moving in the opposite direction, that is, encouraging export by putting all the responsibility on actors who managed the transfer process, whereas producers of waste are completely exempted.

The Convention has particularly focused on the global proliferation of e-waste as well. E-waste has appeared on the agenda of the serial Basel Convention conferences since 2002. In the Strategic Plan for Implementation of the Basel Convention (2000–2010) (formulated at COP.6, 2002), e-waste is identified as one of the priority waste streams to promote partnerships with various businesses and industries. The Mobile Phone Partnership Initiative (MPPI) was launched at the COP.6 by the Basel Secretariat, with well-known globally dominant mobile phone manufacturers (Alcatel, LG, Panasonic, Mitsubishi, Nokia, Philips, Samsung, etc.) After many rounds of discussion and revision, in 2011, the Guidance Document on the Environmentally Sound Management of Used and End-Of-Life Mobile Phones, each chapter of which respectively established general technical guidelines for the collection, transboundary transfer, refurbishment, and materials recovery and recycling of the end-of-life mobile phones, was finally adopted at the COP.10. The MPPI program encourages parties of the Basel Convention to participate voluntarily in the recycling of waste mobile phones. In 2006, the matter of e-waste was a major point of discussion in COP.8 (the topic of the plenary). The COP.8 also announced the Nairobi Declaration on the environmentally sound management of e-waste. The declaration points out several general directions of possible solutions, such as supporting the transfer of available state-of-the-art processing technology of e-waste from developed countries to developing countries; encouraging green and clean technologies and the design of electrical and electronic products; fostering restricted national legislation and firm enforcement on producers’ and traders’ responsibilities and take-back and recycling schemes. In 2008, the program of Partnership for Action on Computing Equipment (PACE) was launched by COP.9 (decision IX/9), to draw on the experience and principles of the MPPI program, to promote the environmentally sound management of waste computing equipment. Similar to the MPPI, it produces a Guidance Document, each chapter of which produces general guidance on the transboundary movement, testing, refurbishment and repair, and materials recovery and recycling of computing equipment. The final document was submitted to COP.13, 2017 and its influences remain yet to be seen. However, similar to MPPI, the program only produces some general principles, without the legal force of constraint.

The heterogeneity of e-waste makes the articles with reference to the regulatory control of transboundary transfer of e-waste rather ambiguous and contradictory. Waste has been classified into two categories in the Basel Convention, namely, “hazardous wastes” (Annexes I, III, VIII, and IX) and “other wastes” (wastes collected from households and incinerator ash, Annex II). Article 1 defines hazardous waste as: (a) Wastes that belong to any category contained in Annex I, unless they do not possess any of the characteristics contained in Annex III; and (b) Wastes that are not covered under paragraph (a) but are defined as, or are considered to be, hazardous wastes by the domestic legislation of the Party of export, import or transit. (Basel Convention, Article 1).

The Basel Convention is only applicable to hazardous wastes. Nevertheless, unlike other types of hazardous waste, e-waste belongs to both “hazardous wastes” and “other wastes” collected from households. The decisions at COP.4 further complicate the regulation of internal e-waste transfer, as it provides a footnote at the end of Annex I to the original text, as follows: (a) To facilitate the application of this Convention, and subject to paragraphs (b), (c) and (d), wastes listed in Annex VIII are characterized as hazardous pursuant to Article 1, paragraph 1 (a), of this Convention, and wastes listed in Annex IX are not covered by Article 1, paragraph 1 (a), of this Convention.

Since then, wastes on List A (Annex VIII) are brought under the regulation of transboundary movement, whereas wastes on List B (Annex IX) are exempt, despite them being identified by the Convention as hazardous wastes. The problem is that e-waste appears on both lists A and B. Many entries in Annex VIII (from A1010 to A1190, A 2010 in Annex VIII) and Annex IX (from B1010 to B1250) are related to e-waste. For instance, many elements of e-waste appear in both entries A 1180 and B 1110 (as seen in Table 3). Yet, the physical and chemical constitution of e-waste is very complex, and different elements are usually articulated or blended, making it highly labor-consuming to separate them.

### 4.2. E-Waste Legislation in Europe and the U.S.

As should be seen from the above analysis, the Basel Convention has many loopholes and contradictory articles and only establishes general or voluntary guidelines. As a result, it, as with many international agreements, largely relies on national states to formulate regulations. It is not a self-executing accord and can only exert robust influences, with member countries formulating auxiliary regulations as a precondition. Yet, at the regional or national level, only four of seven individuals are covered by national e-waste legislation [18], which per se, are frequently contravened in reality. A distinct national difference is observed in e-waste regulations. European countries have formulated and promulgated a series of laws to preclude exporting e-waste to developing countries. The EU Waste Shipment Regulation (WSR) and Waste Electrical and Electronic Equipment Directive (WEEE Directive, hereafter) are two important EU laws that were established to counter illegal e-waste trade [33]. The WEEE Directive has brought the Extended Producer Responsibility (EPR, hereafter) into e-waste regulations in European countries. The EPR is an environmental governance philosophy that aims to expand producers’ responsibility, from only the production phase to cover the user phase and end-of-life phase, based on the principle of polluter-pays. The WSR explicitly defines exporting e-waste to other countries as environmental crime. Despite this, a portion of e-waste is illegally exported from Europe every year, in many cases connected with criminal groups [11,33]; these regulations, in part, do have effects on restricting transnational e-waste flows.

In contrast to European countries, legislations in the largest e-waste exporting the country-the U.S. is deliberately indulging the actions of shipping its domestic e-waste to other countries, with its e-waste regulations manipulated by state power to serve its own environmental and economic interests. Although it is among the first signers of the Basel Convention, the US Senate has yet to ratify it. Considering the astonishing annual generation of e-waste in the US, the ratification of the Convention can bring great pressure on its environment. US laws have made many exemptions for e-waste. In the US legislations, most e-wastes (CRTs are partly excluded) are not recognized as “hazardous wastes.” If e-waste was defined as “hazardous waste,” it should be brought under the regulation of US Resource Conservation and Recovery Act 1976. Instead, it is identified as “special waste” (similarly in Canada’s national regulations) and is exempt from its domestic hazardous waste regulations [1,34,35]. Without the cost of breaking laws like EU countries, vast amounts of US e-waste have been traded to developing countries (mainly China) for decades. However, this is contradictory to the Convention, which stipulates that “A Party shall not permit hazardous wastes or other wastes to be exported to a non-Party or to be imported from a non-Party” (Basel Convention, Article 4.5, page 22). These variegated geopolitical relationships have partly shaped the trajectories of the global e-waste flows.

## 5. China’s Regulatory Frameworks on E-Waste

### 5.1. China’s Contradictory Legislation on E-Waste

Many scholars argue that the lack of sound regulations and laws is the main reason for e-wastes being transported into China. In fact, the Chinese government has long been aware of the potential environmental and health consequences related to informal e-waste recycling [13]. Nevertheless, the state’s attitude towards e-waste recycling had been rather equivocal and self-contradictory until recent years (Table 4).

China signed the Basel Convention on 22 March 1990, and was among the earliest supporters of the prohibitions on the North-to-South transfer of toxic waste. Since the Convention came into force, China has issued a series of regulations with reference to waste import. In 1996, China issued a range of environmental regulations on waste. China’s Act on Prevention and Control of Solid Waste Pollution was put into effect on 1 April 1996. According to Article 25, wastes that cannot be used as raw materials are prohibited from being imported into China, and those that can be used as raw materials are classified into two categories (i.e., restricted import and unrestricted import), to be managed under the Catalogue of Solid Waste Restricted to Import as Raw Materials and the Catalogue of Solid Waste Not Restricted to Import as Raw Materials. Based on this Act, the Interim Provisions on the Administration of Environmental Protection on Wastes Import was jointly issued by multiple ministries of China’s State Council in the same year. Annex 1 of this act is the Catalogue of Solid Waste Restricted to Import as Raw Materials, which contains nine categories of waste, among which the seventh category is “miscellaneous waste hardware, electrical and electronic equipment”. As a result, e-waste is referred to as the seventh category of waste in China’s legislation. This catalogue is the basis for many later policy revisions and is the key policy document for laying down the overarching framework for managing waste import in China. Based on this document, e-waste has been brought into the declaration and inspection system of the state’s customs office as the Seventh Category of Waste that is under restricted import. Only importers with the state’s Certificate of Waste Import Approval can import e-waste into China.

Nevertheless, in 2000, China’s State Environmental Protection Agency (hereafter, SEPA) issued the Notification on Issues Associated with the Import of the Seventh Category of Waste (the Notification, hereafter). This highly brief notification is the first official ban on the import of most types of e-waste. However, a great leeway for importing of e-waste has been left by this notification, as it implies that e-waste can be imported in the name of “copper or aluminium recycling.” The Notification contains two seemingly paradoxical provisions. On the one hand, it prescribes that from 1 April 2000, the import of waste electrical appliances, including TVs, refrigerators, air conditioners, microwaves, computers, monitors, CRTs, digital duplicators, electric cookers, video game consoles (except processing trade), and home telephones, are no longer allowed by the customs office. On the other hand, it also stipulates that all the future allowed import of waste electrical appliances, hardware, electric wires, and cables, should be marked as “recycling copper or aluminium” beside the item “Name of Import Waste” in applying for the Certificate of Waste Import Approval. This self-contradictory statement has created a major loophole for the regulation of e-waste import and is largely responsible for the salient informal status of e-waste recycling in China. In practice, e-waste import, in many cases, is at the interface/grey areas between illegal smuggling and legal import. Even judicial authorities in China are wrestling with this legal ambiguity. For example, in the trial of an e-waste import case in Jinzhou City, Liaoning Province, in 2003, the local court also needed to consult the SEPA about whether the import of computer cases should be considered legal [36]. Many waste brokers transported e-waste into China’s territory by faking, disguising, or concealing the true content and purpose of import. For instance, many waste brokers or metal importers possessing a Certificate of Waste Import Approval (or using others’ certificates) mix e-waste with other types of hardware or metal scraps to “smuggle” it into China. When e-waste is mixed with other types of metal scraps, whether the practice is smuggling is highly debatable, given that the import of mixed-metal scraps is legal. Thus, the legal punishment of e-waste import is merely arbitrarily imposed in some cases.

### 5.2. The State’s Futile Formal Schemes to Compete with the Informal Sector

In addition to e-waste import regulation, the state has long been making enormous efforts to foster the development of formal large-scale recycling enterprises to compete with the informal sector. In 1996, China’s SEPA designated 460 enterprises and certified them as pilot enterprises that could engage in the import or disposal of the seventh-category of waste [14]. In theory, only these state-issued enterprises were allowed to import e-waste. However, these enterprises were not necessarily equipped with qualified technologies or equipment to conduct e-waste recycling in environmentally sound ways. The boundary between formality and informality is highly difficult to distinguish within these types of economic activities. The so-called licensed enterprises were also classified by the state into three levels (levels A, B, and C) based on their scale, facility conditions, and management levels. Enterprises with larger-scale could secure more support from the state. Furthermore, (in)formality is perpetually in influx. After e-waste containers enter China, these so-called formal enterprises commonly re-sold the imported waste to the informal sector rather than process it by themselves. Given that the SEPA has officially banned the import of most e-waste, these certified enterprises also lost the qualifications to import most types of e-waste in 2000. This situation echoes the idea that a constant dialectic occurs between the informal and formal sectors [37]. Based on the Notification, the categories of e-waste listed in the Notification were included to the Catalogue of Prohibited Import Waste issued by the SEPA and other state agencies in 2002.

In the next year, four national schemes collaborated with four of China’s electronics magnates (Dadi in Hangzhou, Huaxing in Beijing, Haier in Qingdao, and Datong in Tianjin), which were initiated by China’s National Development and Reform Commission to occupy the market with formal destruction enterprises. Unfortunately, these four formal enterprises failed the mission with their production lines and operations shut down before long. According to the NDRC’s statistics, from 2003 to 2007, only 191.9 thousand units of sijiyinao in total were collected by these national pilot projects [38]. The amount was rather small in comparison with the hundreds of millions of units of annual e-waste generated in China. Formal plants were commonly struggling with finding e-waste sources [24,39], whereas informal enterprises had a very stable and abundant supply. Informal e-waste collectors were more competent than formal counterparts, given their extensive collection categories, services convenience and flexibility (e.g., door-to-door service), accessibility, and compensation amount for customers [9,40]. Thus, informal backyard e-waste destruction workshops were generally making good profits, whereas most formal plants were frequently losing money [41].

### 5.3. China’s New E-Waste Regime

China’s e-waste regime has been greatly restructured within the past decade. The import of internationally produced of e-waste is banned in the real sense, with the recent tightening up of waste import policy. The sector of e-waste recycling has now mainly shifted to deal with the domestic market. In contrast with previous situations, formal enterprises are processing a large proportion of e-waste, while the collection of e-waste is still relying on street hawkers (Table 5).

Since 2009, various national regulations on e-waste processing have been issued. In 2009, the Promotion Action for Circular Economy was enacted, which introduced the EPR principle into China’s regulatory framework on handling the waste. Internationally, the EPR is widely believed to be a key legislation tool for the governance of e-waste and has been integrated into many countries’ legislation systems. Based on the EPR principle, E-waste Recycling and Disposal Directive (E-waste RDD, hereafter), which was formulated referring to the WEEE Directive in the European Community, was passed in 2008 and came into force in 2011. E-waste RDD is a crucial national regulation that promotes the formalization of e-waste recycling in China. On the one hand, it proposes to impose funding from manufactures to subsidize formal e-waste recycling activities. This goal has been accomplished since the formulation of the Regulations on the Imposition and Usage of E-waste Disposal Fund as its supporting law. Since July 2012, electronics manufacturers have to pay approximately CNY 7–13 for the recycling of each of their products [42]. Formal (state-registered) e-waste recyclers can apply for subsidies of about CNY 85, 80, 35, 35, and 85, respectively, for recycling a TV, a refrigerator, a washing machine, an air conditioner, and a computer, respectively (ibid.) On the other hand, according to the E-waste RDD, e-waste recyclers have to apply for a state-issued license for operating the business of e-waste destruction and disposal. Therefore, all informal recycling activities are now explicitly defined by state law as “illegal.” By 2015, 109 certified enterprises were operating in China. Between 2009 and 2011, large portions of e-wastes were directed to the channel of formal collection. In response to the falling exports because of the 2008 global economic crisis, China began to implement the macroeconomic policy of stimulating domestic demand. To promote the domestic purchase, a “Home Appliance Old for New Rebate Program” was launched by the Chinese government in August 2009 (first in 9 pilot cities for a year, then extended to over 28 provinces in 2011). Formal e-waste collection and dismantling enterprises were integrated into this program for stimulating home appliance consumption. The program involved 1137 sales corporations, 1116 collection enterprises and 105 dismantling enterprises designated by the state [43]. Under this program, consumers can surrender their obsolete home appliances to the government-designated e-waste collectors to obtain a voucher, so that they can then use it for applying for a subsidy to purchase a new electronics product (only applicable to five types of household appliances, i.e., TVs, refrigerators, air-conditioners, washing machines, and computers (including laptops and tablets). The subsidy is worth 10% of the original price of the new electronics product. As a result, substantial amounts of e-waste that consumers previously sold to street vendors or simply kept at home were diverted to formal e-waste recyclers [44,45]. By the end of April 15, 2011, approximately 46.6 million units of obsolete home appliances had been collected by the certified collectors [24]. Nevertheless, after the policy and corresponding subsidies ceased by December 2011, formal collectors began to face the previous problem of lack of supply because of the e-waste flowing into street vendors again. The certified 105 dismantling enterprises returned to the situation of depending on the informal sector for e-waste collection, despite the final processing being increasingly handled by the formal dismantling enterprises.

In 2012, the e-waste pollution in Guiyu was identified by China’s SEPA as one of SEPA’s top three battles. After that, a full-scale formalization scheme was initiated by the Guangdong provincial government, with the compilation of the “Comprehensive Remediation Scheme of Guiyu E-waste Pollution” 2013. Based on the plan of the Scheme, the Guiyu Circular Economy Industrial Park covering around 82.4 acres of land, was built and put in use at the end of 2015. Some large formal destruction enterprises (mainly, TCL and China Energy Conservation and Environmental Protection Group) were brought into the park. According to the Interpretation of the Supreme People’s Court and the Supreme People’s Procuratorate on Several Issues concerning the Application of Law in the Handling of Criminal Cases of Environmental Pollution [46] implemented in 2017, the storage and disposal of e-waste over three tons are illegal. All the e-waste recycling workshops in Guiyu, except those conducting the classification of plastics with little pollution, were shut down unless they moved to the industrial park. Nevertheless, as a result of the high cost of rent, water, electricity, pollution charge, management fee, tax, etc. (more than 10,000 RMB per year) in the park, most e-waste recycling shops could only choose to close down under the state’s suppression, with only less than 1000 workshops left.

Despite the enormous raw materials provided by e-waste recycling during the rise of China’s manufacturing, Chinese governments embarked on the tightening up its waste import recently, pertaining to its skyrocketing domestic waste generation and growing material supply capability. As mentioned earlier, substantial e-waste was imported into China in the name of “recycling of copper or aluminium” under the Seventh Category of Waste. Nevertheless, the import of the Seventh Category of Waste had been tightened up since 2017 and was completely banned at the end of 2018 [47]. In July 2017, China’s State Council released the Implementation Plan on Banning Imports of Foreign Garbage and Advancing the Reform of the Solid Waste Import Administration System. The plan was known as China’s strictest policy on waste import in history. The import of 24 varieties of solid waste is banned by this policy, including plastics, which have significant impacts on the seven specialized plastics processing villages in Guiyu. In December 2018, China’s Ministry of Ecology and Environment (the new name of the SEPA) released the adjustment of the Catalogue of Imported Waste, and the Seventh Category of Waste was moved from the Catalogue of Solid Waste Restricted to Import as Raw Materials to the Catalogue of Solid Waste Forbidden to Import into China. Since 2017, Guiyu e-waste recyclers have faced increasing difficulties in obtaining the supply of imported e-waste. Alongside the implantation of this suite of policies and regulations, the global e-waste hub Guiyu gradually withered away, with only a small portion of e-waste economies left.

## 6. Conclusions

This paper offers a nuanced analysis of the multi-scalar regulatory frameworks, in which China’s informal e-waste recycling and environmental pollutions are embedded. We argue that the problematic and contentious use of the international and national legislation to regulate e-waste is in part pertaining to the slippery definition of what counts as “e-waste” and its contradictory nature as both resources and pollutants.

The plethora of documents of the Basel Convention has left a multitude of loopholes, contradictions and ambiguous articles, which constitute the major source of the informality of the transnational trade of e-waste. The heterogeneity of e-waste and its complex compositions produce many impasses for applying the legal provisions of the Basel Convention. E-waste appears on both of the catalogues of waste categories that are allowed to be traded and those not allowed in the Convention, partly due to the slippery definition of “e-waste”. The Convention stays at a rather general level and relies on the subordinate regional, national laws, or regulations to put it into effect. Nevertheless, the Basel Convention has facilitated the production of regional and national legislations. These subordinate regulations show a prominent geographical differentiation at the global level. Influenced by the Basel Convention, the EU WEEE Directive has lessened the export of e-waste generated in Europe to China to a certain level. By contrast, the deliberate circumvention of the Basel Convention in the US regulatory framework of e-waste opens the door for shipping e-waste to China.

At the national scale, the ambiguities and contradictions in the Basel Convention have been passed on to and shaped China’s national e-waste regulatory frameworks. The territorialization of globally produced e-waste in China is not a result of the absence of relevant regulations and laws. The rise of e-waste recycling in Guiyu is largely pertaining to China’s equivocal legislation, the state’s ambivalent attitude, and futile formal schemes. The contradictory nature of e-waste, as resources/raw materials and pollutants/hazardous simultaneously, leads to the state’s bewildering attitude toward a stern ban on e-waste import. China’s legislation concerning e-waste had not been seriously implemented until recently and penalties are randomly and arbitrarily enforced. E-waste import in many cases was conducted in acquiescence, but was regarded as illegal smuggling in other times. Some later policy revisions also increase the complexity of e-waste regulation. As a result, e-waste importation had fallen into a grey area of regulations for a long time. Even judicial authorities in China sometimes struggled to understand the legal status of e-waste import in making forensic decisions. The formal e-waste destruction schemes initiated by Chinese governments failed to compete with the informal sector as a result of their unstable e-waste supply and informal sectors’ wide range of advantages (e.g., accessibility, flexibility, and higher price).

Yet, the previous e-waste regime has been greatly restructured by a suite of policies and laws enacted since 2009. A new e-waste regime has emerged in the past few years. E-waste economies are now targeting the recycling of domestic generation, while excluding globally imported e-waste, with the tightening up of China’s waste import policies. Despite the continuing reliance on the informal sector for collection (except the ensuing three years after the 2008 economic crisis), formal enterprises, granted with substantial subsidies raised from taxes from electronics manufacturers, are now playing an increasingly significant role in the final destruction and disposal of e-waste. E-waste destruction has shifted from informal economies to a sector now explicitly defined by current regulations as illegal. With the tightening up of waste import policies and government’s suppression of informal economies in Guiyu, the former global e-waste recycling hub, to a great extent, has withered.

## Figures and Tables

**Table 1:** Global generation of e-waste.

Countries	E-Waste Generation in 2014 (Million Metric Tons)	E-Waste Generation in 2016 (Million Metric Tons)
**Global**	40.00	44.70
**U.S.**	7.07	6.30
**China**	6.03	7.20
**Japan**	2.20	2.10
**Germany**	1.80	1.90
**India**	1.64	2.00
**U.K.**	1.51	1.60

Data source: United Nations University report [1,18].

**Table 2:** Annual generation of waste home appliance in China.

Year	2017		2018	
Categories	Quantity (1000 units)	Weight (1000 tons)	Quantity (1000 units)	Weight (1000 tons)
**TV sets**	30,650	797	48,176	853
**Refrigerator**	16,880	608	20,647	970
**Washing machine**	16,990	356	20,248	435
**Air conditioner**	16,820	572	31,491	1203
**PC**	18,930	284	30,344	607
**Total**	100,270	2618	150,906	4068

Source: (CHEARI, 2019).

**Table 3:** Basel Convention on e-waste.

Entries	Content
**A1180**	Waste electrical and electronic assemblies or scrap containing components such as accumulators and other batteries included on list A, mercury switches, glass from cathode-ray tubes and other activated glass and PCB-capacitators, or contaminated with Annex I constituents (e.g., cadmium, mercury, lead, polychlorinated biphenyl), to an extent that they possess any of the characteristics contained in Annex III (note the related entry on list B 1110)
**B1110**	Electrical and electronic assemblies: Electronic assemblies consisting only of metals or alloys Waste electrical and electronic assemblies or scrap (including printed circuit boards), not containing components such as accumulators and other batteries included on list A, mercury-switches, glass from cathode-ray tubes and other activated glass and PCB- capacitors, or not contaminated with Annex I constituents (e.g., cadmium, mercury, lead, polychlorinated biphenyl) or from which these have been removed, to an extent that they do not possess any of the characteristics contained in Annex III (note the related entry on list A A1180) Electrical and electronic assemblies (including printed circuit boards, electronic components and wires) destined for direct reuse, and not for recycling or final disposal

Source: Excerpts from the Basel Convention.

**Table 4:** China’s key legislation on e-waste before the new millennium.

Year	Name of Regulations	Key Function and Influences on E-Waste Management
1996	Act on Prevention and Control of Solid Waste Pollution in China	As a national regulation in response to the Basel Convention; Article 25 categorizes waste import into “restricted import” and “unrestricted import”
1996	Interim Provisions on the Administration of Environmental Protection on Wastes Import Catalogue of Solid Waste Restricted to Import as Raw Materials, which is Annex 1 of the above Interim Provision.	Bring e-waste under China’s legislation as the 7th category of waste; Establishing the declaration and inspection system for e-waste import
2000	Notification on Issues Associated with the Import of the Seventh Category of Waste	Ban on the import of most categories of e-waste (yet leaving loopholes for e-waste mixed with meal scraps to be imported)

**Table 5:** China’s key legislation on e-waste in the recent decade.

Year	Name of Regulations	Key Function and Influences on E-Waste Management
2009	Circular Economy Promotion Law of the People’s Republic of China	Introducing the EPR (Extended Producer Responsibility) principle into China’s regulatory frameworks on waste recycling
2009–2011	Home Appliance Old for New Rebate Program	Directing e-waste flows to large formal collectors, which are actually electronic manufacturing magnates in China
2011	E-waste Recycling and Disposal Directive	Proposing to impose funding from manufacturers to subsidize formal e-waste recycling activities Explicitly define informal e-waste recycling activities without certificates as illegal
2013	Comprehensive Remediation Scheme of E-waste Pollution in Guiyu Town of Shantou City	Formalize and suppress the informal e-waste economies in Guiyu
2017	Implementation Plan on Banning Imports of Foreign Garbage and Advancing the Reform of the Solid Waste Import Administration System	Ban on the import of waste that can be recycled for raw materials comprehensively Completely cut off the channel for e-waste to be mixed with other metal scraps to be imported into China
